# Case report: Nonsimultaneous bilateral triceps tendon rupture and surgical repair in a healthy dog

**DOI:** 10.3389/fvets.2023.1294395

**Published:** 2024-01-24

**Authors:** Maureen P. Bennett, Gena Silver, Tonya Tromblee, Rickard Kohler, Daniel Frem, Eric N. Glass, Marc Kent

**Affiliations:** ^1^Section of Neurology and Neurosurgery, Massachusetts Veterinary Referral Hospital, Woburn, MA, United States; ^2^Section of Diagnostic Imaging, Massachusetts Veterinary Referral Hospital, Woburn, MA, United States; ^3^Section of Orthopedic and Soft Tissue Surgery, Massachusetts Veterinary Referral Hospital, Woburn, MA, United States; ^4^Section of Neurology and Neurosurgery, Red Bank Veterinary Hospital, Tinton Falls, NJ, United States; ^5^Department of Small Animal Medicine and Surgery, College of Veterinary Medicine, University of Georgia, Athens, GA, United States

**Keywords:** triceps, bilateral, rupture, tendon, MRI, canine

## Abstract

A 7-year-old female spayed Australian shepherd dog was presented for an acute onset of inability to stand. On physical examination, the dog was unable to support weight on the thoracic limbs. On neurological examination, the thoracic limbs had absent hopping and paw placement and reduced withdrawal reflexes bilaterally. The remainder of the neurological examination was normal. The anatomic lesion localized to the C6-T2 spinal nerve roots, spinal nerves, or the named nerves of the thoracic limb, bilaterally. A lesion affecting the ventral gray column of the C6 through T2 spinal cord segments was considered less likely. In an effort to exclude an orthopedic disorder from consideration, radiographs of the shoulders, elbows, and manus were normal. Magnetic resonance imaging of the cervical and cranial thoracic vertebral column was normal. Analysis of synovial fluid from the carpi, elbows, and shoulders were normal. Ultrasonography of the triceps muscle and tendon of insertion revealed bilateral, acute-subacute tears of the tendon at insertion of the triceps muscles, bilaterally. Magnetic resonance imaging of both elbows revealed complete avulsion of the triceps tendons bilaterally. Surgical repair of both tendons was performed using the Arthrex FiberLoop system combined with autologous conditioned plasma soaked in a collagen sponge. Postoperatively, external coaptation was provided using Spica splints for 6 weeks followed by the use of soft padded orthotic braces for an additional 6 weeks. Concurrently, a front support wheelchair was used for 10 weeks postoperative. By 10 weeks postoperative, the dog was able to ambulate without support. To the authors’ knowledge, this is the first report of bilateral triceps tendon avulsion in a dog. Tendon avulsion occurred without a known history of trauma or predisposing metabolic abnormalities. Magnetic resonance imaging provided excellent anatomical detail that aided in surgical repair.

## Background

In dogs, the triceps brachii muscle consists of the long, lateral, medial, and accessory heads, with a common insertion tendon on the olecranon (1). The triceps brachii muscle functions to flex the shoulder and extend the elbow joints (2). Given its function to extend the elbow joint, the triceps brachii muscle is essential for the ability to support bodyweight and ambulate normally. Following triceps brachii muscle or tendon injury, therapeutic intervention is often required for return of function (3). Such injuries most commonly involve the tendon of insertion, although disruption at the musculotendinous junction or avulsion of the tendon at the distal osteotendinous junction may occur (4).

To the author’s knowledge, the English-language published veterinary literature details rupture or avulsion of the triceps brachii tendon in 13 dogs (2–10). Causes of triceps brachii tendon injury include trauma (e.g., falling from height, dog bite, car accident, iatrogenic), unknown, (1–4, 9, 10) and corticosteroid administration (6, 7). The diagnosis is based on the combination of physical examination, radiography, and ultrasonography (4). However, a definitive diagnosis occurs at the time of surgical repair in the majority of cases (4). Magnetic resonance imaging may also provide for a definitive diagnosis of tendon injury with the added benefit of providing aid in treatment decisions for surgical repair techniques (1, 3). With complete tendon rupture, treatment almost always entails surgical repair as conservative therapy with external coaptation and restriction of movement alone rarely results in healing and restoration of function (2). Post-operative immobilization with external coaptation for 6–10 weeks is used to prevent failure of the primary surgical repair (2).

The case report herein describes a previously healthy dog that developed non-ambulatory thoracic limb lameness due to non-simultaneous, complete avulsion of both triceps brachii tendons. An inciting cause was not identified. Magnetic resonance imaging established a definitive diagnosis. Primary surgical repair was performed using the Arthrex FiberLoop system with an autologous conditioned plasma-soaked collagen sponge. To the authors’ knowledge, this is the first report of bilateral triceps brachii tendon avulsions.

## Case description

A 7-year-old, 22 kg spayed female Australian shepherd dog was evaluated at Massachusetts Veterinary Referral Hospital for a history of progressive difficulty walking. Five days prior to evaluation, the dog developed weight-bearing lameness of the right thoracic limb after being outside unsupervised. Treatment by the referring veterinarian consisted of carprofen (1.7 mg/kg *per os* [PO] q12 h [Rimadyl, Zoetis, Kalamazoo, MI, United States]) and gabapentin (13.6 mg/kg PO q12 h [Neurontin, Pfizer Inc., NY, United States]). Despite treatment, the lameness progressed over 2 days to non-weightbearing. One day prior to presentation, the dog vocalized outside unsupervised and was unable to walk. The dog had no prior medical history and was up to date on vaccinations and preventatives.

At presentation, the physical examination was unremarkable. The dog did not seem painful upon palpation or manipulation of the cervical spine, the long bones, or joints of either forelimb. Ranges of motion in the neck and joints of the thoracic limbs were within normal limits. On neurological examination, the dog had a normal mentation and was unable to stand and walk. Paw placement and hopping reflex was absent in both forelimbs. When hopping was performed, the dog would roll into lateral recumbency or fall forward. The withdrawal reflexes were reduced in the thoracic limbs. The dog held her thoracic limbs in a flexed position at the level of the elbows and carpi. The postural reactions and spinal reflexes in the pelvic limbs were unremarkable on examination. The remainder of the neurological examination was considered unremarkable. Based on the neurological examination findings, the anatomic diagnosis was consistent with a lesion affecting the C6 to T2 spinal nerve roots, spinal nerves, or named nerves of the thoracic limbs. A less likely consideration was dysfunction of the ventral gray column of the C6 to T2 spinal cord segments as the pelvic limb postural reactions were normal.

Complete blood count revealed lymphopenia (lymphocytes 340/ul; reference range 830 to 4,910/ul). Serum chemistry was normal. Thoracic radiographs revealed a diffuse bronchointerstitial pattern consistent with age-related changes. Radiographs of both shoulders, elbows, carpi, and manus were normal.

Under general anesthesia, magnetic resonance imaging (MRI) of the vertebral column from the first cervical vertebra through the fourth thoracic vertebra was performed using a 1.5-Tesla MRI (Signa HDxt, GE Healthcare, Waukesha, WI, United States) and a phased array spine coil. The following multiplanar sequences were obtained: sagittal and transverse T2-weighted (T2W) and T1-weighted (T1W) images, and dorsal short tau inversion recovery (STIR) images. Following 0.1 mmol/kg IV intravenous administration contrast medium (Gadavist, Bayer Healthcare Pharmaceuticals, Whippany, NJ, United States), sagittal and transverse T1-weighted images also were obtained. No abnormalities were noted in the vertebral column, spinal cord, spinal nerves with the exception of dehydration of the nucleus pulposus of the C6-C7 intervertebral disc. Analyzes of synovial fluid obtained from the right and left carpus, elbow, and shoulder joints were consistent with mild mononuclear inflammation consistent with degenerative joint disease.

Following the absence of diagnosis on MRI of the cervical vertebral column, repeat examination of the thoracic limbs disclosed subtle, firm, painful swellings proximal to the olecranon along the course of the tendon of insertion of the triceps brachii muscles, bilaterally. Just distal to the swelling, small palpable defects in the triceps brachii tendon were appreciated bilaterally.

Ultrasonographic assessment of the distal triceps brachii muscle and tendons of insertion was hindered by swelling and markedly heterogenous hyperechoic tissue presumed to represent hemorrhage and cellulitis. A clearly delineated tendon of insertion could not be appreciated in either limb. However, approximately 2 cm proximal each olecranon, remnant segments of the triceps tendon were thickened and hyperechoic with altered fiber pattern and several hypoechoic, sagittal defects consistent with disruption of tendon fibers. Ultrasonographic findings were consistent with bilateral, acute to subacute, partial to full thickness tear of the triceps tendons near the insertion site. To further characterize the ultrasonographic abnormalities of the triceps muscle and tendon of insertion as well as guide potential surgical intervention, an MRI of the elbows was performed.

**Figure 1:**
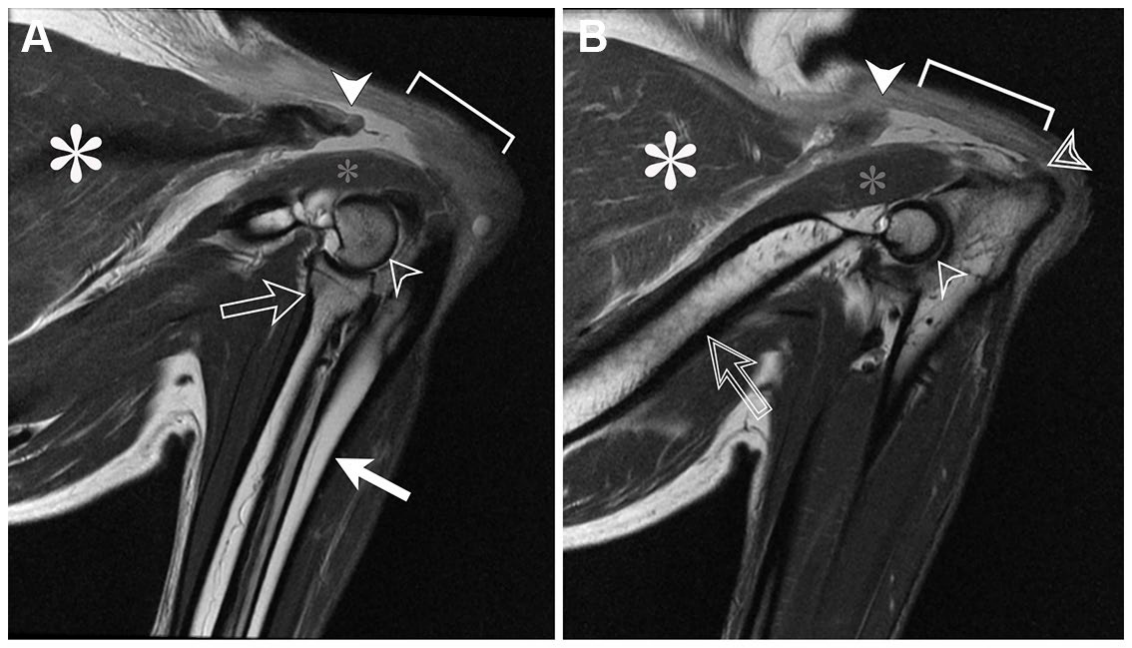
Sagittal proton density magnetic resonance images of the right **(A)** and left **(B)** elbows positioned in flexion; the brachium is directed to the left side of the images and the antebrachium is directed toward the bottom of the images. The triceps brachii muscle (white asterisk) tapers distally toward the area of the musculotendinous junction where it is abruptly truncated (white arrowhead). Distal to this, the normally hypointense tendon of insertion is absent (braces). Instead, the tendon is retracted proximally toward the muscle. A remnant tendon stump remains at the olecranon tuber (**B**, double lined arrowhead). The anconeus muscle (small gray asterisk) appears normal. Other structures include the lateral humeral condyle (open arrowhead), radial head and neck (**A**, open arrow), ulna (**A**, white arrow), and humerus (**B**, doubled lined arrow).

**Figure 2:**
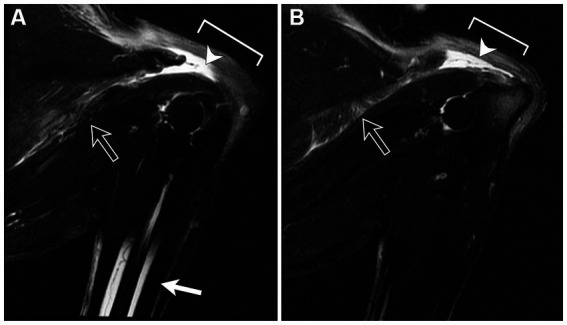
Sagittal fat-saturated T2-weighted magnetic resonance images of the right **(A)** and left **(B)** elbows positioned in flexion. There is marked increase in signal intensity in the region of the triceps tendon with proximal retraction of the tendon stump. Additionally, there is increased signal within the accessory head and regional fascia (open arrow) consistent with edema. Incomplete medullary fat suppression is noted in the diaphysis of the radius and ulna (**A**; white arrow).

Under general anesthesia, MRI of the elbows was performed using the same MRI unit and phased array spine coil. The elbows were positioned in flexion. Sagittal and transverse T1W, T2W using chemical shift fat suppression, and proton density-weighted (PD) images of both elbows were obtained. Both triceps brachii tendons of insertion were completely avulsed distal to the musculotendinous junction (Figures 1, 2). On the right side, there was proximal retraction of the triceps muscle and undulation of the remnant tendon. Distal to the avulsion, a 0.7 cm long segment of tendon remained attached to the olecranon tuber. On the left side, a 1.7 cm long segment of tendon remained attached to the olecranon tuber with a smaller retraction gap between the muscle and tendon. In both remnant tendon segments, there was a linear increase in T2W and PD signal consistent with intrasubstance fibrillar tear. Thin, filamentous strands of tendon fibers projected from both ends of the tendon segments. In both triceps brachii muscles, there was increased T2W signal within the accessory head and adjacent fascia and increased T2W and PD signal encircling the triceps tendon insertion consistent with edema. The other head of the triceps brachii muscles were unaffected. The remaining periarticular soft tissues were normal. Both elbows lacked degenerative changes or joint effusion.

The MRI findings were consistent with a complete avulsion of right triceps brachii tendon near the olecranon attachment and complete avulsion of left triceps brachii tendon at mid-portion of the tendon.

**Figure 3:**
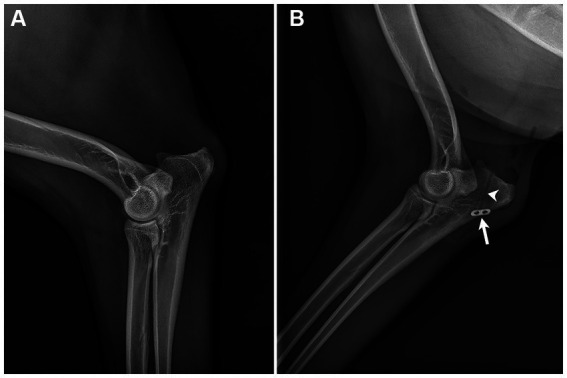
Preoperative **(A)** and post-operative **(B)** lateral radiographs of the right elbow. No osseous or notable soft tissue abnormalities are seen in the pre-operative image. The 2.0 mm bone tunnel (arrowhead) and anchor (arrow) securing the distal end Arthrex FiberLoop are shown **(B)**.

The dog underwent single-session, bilateral surgical repair of the triceps brachii tendons. Identical surgical approaches were utilized in both limbs. Briefly, a lateral approach to the elbow was used. The subcutaneous tissues were sharply dissected to reveal the completely disrupted triceps brachii tendon. The tendon was dissected from the surrounding fascia. The distal end of the proximal segment of the disrupted tendon was sharply debrided until normal appearing fibers were visible. An Arthrex FiberLoop suture (Arthrex Inc.; Naples, FL, United States) was used with a loop suture technique as previously described (11). Six loops were placed in the proximal segment of the disrupted tendon. A bone tunnel was created in the olecranon tuber using a 0.045″ k-wire and 2.0 mm cannulated drill bit. The suture was then passed through the bone tunnel, pulled to approximate the tendon to the olecranon without a gap, and secured with a stainless steel Proximal Tenodesis Button (Arthrex Inc.; Naples, FL, United States). Epitendinous sutures of 0-PDS (Polydioxanone, Johnson and Johnson, New Brunswick, NJ, United States) were placed along the caudal aspect of the tendon. Two milliliters of autologous platelet rich plasma was prepared as previously described (11). A collagen sponge (CollaVET Sponge, Collagen Matrix INC; Oakland, NJ, United States) was soaked in the PRP and wrapped around the site of repair to improve tendon healing (12–14). The surgical site was closed routinely. Appropriate trajectory of the bone tunnel and placement of the steel button were confirmed on postoperative radiographs (Figure 3).

Postoperatively, Spica splints were applied with the elbow in approximately 150^0^–160^0^ degree extension bilaterally. A front support cart (Walkin’ Pets, Amherst, NH, United States) was fitted to the dog to allow mobility and prevent lateral recumbency for extended periods. The dog was discharged 3 days post-operative on gabapentin (13.6 mg/kg PO q8-12 h [Neurontin, Pfizer Inc., NY, United States]), trazodone (4.5 mg/kg PO q8-12 h [Desyrel, Pragma Pharmaceuticals, Locust Valley, NY, United States]), and carprofen (1.7 mg/kg PO q12 hr. [Rimadyl Zoetis, Kalamazoo, MI, United States]) for 10 days. The Spica splints were revised twice a week at which time, the joints were put through range of motion and the integrity of the surgical repair of the tendons were assessed by manual flexion of the elbows and supervised weight bearing of the limbs without external coaptation. While splinted, the dog was allowed to walk in the front support cart with the thoracic limbs suspended to allow moderate weight-bearing. The Spica splints were maintained for 6 weeks. At 3 weeks post-operative, superficial pyoderma developed along the incision of the right medial elbow. Aerobic culture grew *Staphylococcus pseudintermedius*. The dog was treated with cephalexin (22 mg/kg PO q12 hr. [Keflex, Pragma Pharmaceuticals, Locust Valley, NY, United States]) for 28 days which resolved the infection. After 6 weeks in Spica splints, the dog was transitioned to soft support elbow orthotic braces (DogLeggs, LLC., York, PA, United States) while still utilizing the support cart. Use of the support cart was discontinued at 10 weeks after surgery and the dog was able to walk without support. At 18 weeks post operative, the dog was walking without orthotics and no external support with a normal gait.

## Discussion

The case herein initially presented a diagnostic challenge. Given the bilateral presentation and the initial lack of conspicuous pain or physical evidence of a muscular or orthopedic abnormalities combined with not having witnessed a traumatic incident, triceps brachii tendon injury was not considered. Moreover, the abnormal postural reactions and decreased withdrawal reflexes in the absence of elicitable pain in the limbs raised suspicion for an underlying neurological disorder. In retrospect, the abnormal postural reactions in the dog in this report likely reflected a mechanical inability to support weight rather than true neurologic dysfunction. In people, misdiagnosis of triceps rupture also commonly occurs as a consequence of a low index of suspicion, pain that may prevent thorough evaluation, and tissue swelling that may mask a palpable defect in the triceps brachii tendon (15, 16).

As in people, triceps brachii tendon injury is rarely encountered in veterinary medicine. While physical examination and radiography often support the diagnosis, ultrasonography and MRI substantially contribute to the definitive diagnosis. In humans, MRI is the gold standard imaging modality for the diagnosis of triceps brachii tendon injury. MRI allows not only for identification of partial and complete tendon rupture but may also enable evaluation of the integrity of the superficial or deep portions of the tendon, which ultimately helps guide treatment decisions and surgical strategy in humans (17). Frequently, recommended treatment for acute, complete rupture of the triceps tendon in dogs is surgical correction followed by external immobilization of the joint (3). Without MRI in most of the previously reported cases of triceps rupture in dogs, the degree of tendon rupture (partial vs. complete) was only able to be accurately determined at the time of surgery (4). In the present case report, MRI enabled identification of a complete tendon rupture which was not appreciated with ultrasonography due to surrounding soft tissue swelling and edema.

MRI is an underutilized modality in veterinary medicine for evaluation of musculoskeletal structures due to the need for general anesthesia and associated cost. However, it is less operator-dependent than ultrasonography and can provide greater information regarding the extent and chronicity of musculotendinous injury (18). MRI has been utilized in the evaluation of triceps tendon rupture in dogs (1, 3). In these reports, triceps tendon ruptures were chronic with obvious physical examination abnormalities including painful swellings proximal to the olecranon, pronounced atrophy of the triceps muscles and palpable defects in the triceps tendon of insertion (1, 3). In the one report that documented neurological assessment, neurological examination was normal (1). Given the conspicuity of triceps tendon pathology on physical examination, MRI evaluation of the elbow in previous reports likely provided confirmation of tendon rupture (1, 3). In contrast, MRI evaluation in the present case served to provide a definitive diagnosis of complete tendon rupture wherein physical examination, radiography, and ultrasonography, to a lesser degree, were unable to adequately assess the integrity of the triceps tendon. Although not utilized in the present case, MRI may also be a valuable tool to evaluate postoperative healing of injured tendons and guide progressive re-mobilization and return to activity (9).

Given that the patient in this case was bilaterally affected, there was concern for the amount of stress that would be placed on the surgical repairs throughout healing. The Arthrex FiberLoop was first described for canine calcaneal tendon repair, but was adapted from human tendon repair techniques (11). It had been utilized successfully in previous instances by the authors on multiple calcaneal tendon repairs, and therefore was further adapted to fit this case. The technique is technically easier and faster to perform than other common patterns for ligament repair (e.g., 3-loop pulley, direct apposition). To the authors’ knowledge, there is no biomechanical analysis that compares the FiberLoop technique to alternative suture patterns in dogs.

The low metabolic rate of tendons results in slower healing times compared to other tissue types, with tendons achieving less than 60% of their original strength 6 weeks after repair, and only up to 80% original strength 1 year post-repair in animal models (3). Gradual remobilization allows for steady increases in range of motion of the joint while maintaining support and controlled loading (4, 19). Controlled loading over a tendon repair initiated 3–4 weeks post-repair results in more rapid return of strength when compared to longer immobilization (19). For these reasons, the patient in this case was first placed in a front-support wheelchair with bilateral splints 6 weeks postoperatively, which was considered the time to achieve roughly 50% of tendon strength. It is well known that the complete immobilization of the shoulder and elbow is extremely difficult in domestic animals, therefore we presume that some strain was present throughout healing despite attempts at immobilization. The wheelchair was primarily utilized to assist the owners with mobility during this time. After this period, soft elbow orthotic braces were then utilized for controlled joint loading for another 8 weeks. In this case, splints were utilized not only to provide support but also enable slight range of joint motion and thereby decrease the risk of ankylosis or muscle contracture (20).

Several limitations hinder any broad conclusions from the case reported here. First, this is a single case in which an inciting cause or predisposing factors were not identified. Moreover, tendon fragments were not submitted for histopathologic analysis to investigate for chronicity or an underlying tendinopathy. Despite this, evidence of chronic avulsion at the tendon insertion or significant disuse atrophy of the triceps muscle was lacking on the MRI (3). Notwithstanding these limitations, the present case report suggests that MRI is a useful imaging modality not only to define triceps tendon pathology but also to help guide the need for surgical intervention. In humans, the amount of tendon disruption guides surgical strategy. Although such guidelines are lacking in veterinary medicine, the present case highlights the potential for MRI to exactly mirror visible pathology at surgery and best assess tendon integrity. Additionally, ultrasonography was unable to delineate the extent of the tendon rupture.

## Concluding remarks

To the authors’ knowledge, this is the first case report describing bilateral triceps tendon rupture in a dog. A commercially available suture loop was used for single-staged, bilateral surgical repair combined with autologous platelet rich plasma. Post-operatively, dynamization of external coaptation was achieved through the use of various devices combined with the use of a cart which likely contributed to return to normal function. Physical exam and other imaging modalities underestimated the extent of the triceps tendon disruption whereas MRI was instrumental in identifying complete tendon avulsions. To date, MRI remains underutilized in the evaluation of musculoskeletal disorders in veterinary medicine. In the present case, knowledge of the extent of the injury observed on MRI helped guide the pursuit of surgical repair over a conservative approach. While speculative, greater use of MRI in the evaluation of triceps tendon injury in dogs may lead to the development objective therapeutic guidelines in deciding conservative management versus surgical intervention.

## Data availability statement

The raw data supporting the conclusions of this article will be made available by the authors, without undue reservation.

## Ethics statement

Written informed consent was obtained from the owners for participation of their animal in this study. Written informed consent was obtained from the participant/patient(s) for the publication of this case report.

## Author contributions

MB: Writing – original draft, Writing – review & editing. GS: Writing – original draft, Writing – review & editing. TT: Writing – original draft, Writing – review & editing. RK: Writing – review & editing. DF: Writing – review & editing. EG: Writing – review & editing. MK: Writing – original draft, Writing – review & editing.
